# Editorial: Blood, cerebrospinal fluid, and vascular biomarkers for dementia

**DOI:** 10.3389/frdem.2026.1747825

**Published:** 2026-01-29

**Authors:** Nobuyuki Kobayashi

**Affiliations:** Maintain Brain Inc., Tokyo, Japan

**Keywords:** blood-based biomarkers, cerebrospinal fluid, dementia biomarkers, metabolomics, neuroimaging, proteomics, vascular markers

The Research Topic *Blood, Cerebrospinal Fluid, and Vascular Biomarkers for Dementia* was established to bring together studies that collectively reflect the increasingly multidimensional nature of dementia research. Dementia arises from the interplay of molecular pathology, vascular and metabolic processes, systemic physiology, and age-related structural and functional brain changes. As such, single-modality research cannot sufficiently capture its biological complexity. This Topic aimed to highlight how complementary biomarker domains—blood, cerebrospinal fluid (CSF), vascular indicators, proteomics, metabolomics, and neuroimaging-derived measures—can contribute to more integrated frameworks for early detection, diagnosis, and mechanistic understanding.

Blood-based biomarkers constitute a major theme across the contributions. Several studies validate plasma markers of Alzheimer's disease (AD) pathology, including the performance of P-tau181/Aβ42 in real-world settings (Liu D. et al.). Serum neurofilament light chain is examined in a large national cohort (Meng et al.), while hematologic indices such as the hemoglobin-to-red cell distribution width ratio show associations with cognitive functioning in older adults (Wang et al.). Vitamin D concentration demonstrates a dose-dependent relationship with dementia risk (Huang et al.), and serum albumin appears protective in prospective data from the UK Biobank (Cui et al.). Proteomic profiling reveals early-stage molecular alterations in AD (Wei et al.), and genotype-specific immune–metabolic interactions emerge from work on lipopolysaccharide-binding protein (Romo et al.). These studies illustrate how blood-based biomarkers, spanning neurodegeneration, inflammation, nutrition, and systemic metabolism, can complement established proteinopathy markers.

Vascular and metabolic physiology represent another key dimension of dementia risk. Elevated homocysteine combined with reduced renal function increases the likelihood of post-stroke cognitive impairment (Zhang et al.). Variability in systolic blood pressure predicts cognitive trajectory and AD risk in late life (Li X-L. et al.), while the triglyceride–glucose index shows consistent associations with cognitive impairment across national datasets (Yang et al.). The interaction between hypertension and plasma β-amyloid in shaping cognitive outcomes highlights the modifying effect of vascular load on biomarker expression (Liu Z. et al.). Additionally, hyperuricemia appears to reduce the likelihood of mild cognitive impairment though not dementia (He et al.). Together, these studies strengthen the view that vascular and metabolic dysregulation are central—not peripheral—components of dementia biology.

CSF biomarkers continue to serve as reference standards for AD and other neurodegenerative diseases. Chemiluminescence immunoassays demonstrate reliable differentiation of AD from other disorders (Arendt et al.), while work in familial frontotemporal dementia synthesizes current progress in CSF and plasma markers linked to genetic variants (Guo et al.). Associations between varicose veins and CSF Alzheimer pathology in adults without dementia (Liu M. et al.) suggest potential systemic–central interactions that warrant further exploration. These studies reaffirm the diagnostic value of CSF measures while pointing to broader physiological factors that may shape biomarker profiles.

Neuroimaging and multimodal approaches expand the conceptual scope of this Topic. The DTI-ALPS index, reflecting glymphatic function, enhances prediction of cognitive impairment in Parkinson's disease when combined with renal measures (Gao et al.). BIN1 polymorphisms are linked to hippocampal subfield vulnerability in community-based mild cognitive impairment (Luo et al.). Integrating linguistic features with biomarker data improves early AD screening (Chou et al.), and circadian-related genomic insights offer potential targets for therapeutic modulation (Li Z. et al.). These contributions underscore the importance of linking molecular pathology with behavioral and structural-functional data.

Systemic biomarkers and global perspectives further enrich the Topic. Untargeted urine metabolomics differentiates metabolic states across AD stages (Feng and Zhao), while a bibliometric analysis of normal pressure hydrocephalus charts the evolution of research priorities in the field (Chang et al.). Collectively, these findings emphasize that dementia pathophysiology is distributed across molecular, metabolic, vascular, inflammatory, and structural domains.

Across the 26 contributions, several shared principles emerge. First, minimally invasive biomarkers—particularly blood-based proteins, metabolic indices, and inflammatory markers—are increasingly positioned for scalable population-level screening. Second, vascular and metabolic dysregulation are integral drivers of cognitive decline. Third, multimodal integration across molecular, imaging, and behavioral data is essential for achieving biological coherence and diagnostic precision.

Although the present Topic does not directly evaluate epigenetic biomarkers, regulatory processes such as DNA methylation represent a promising future direction. Many upstream factors highlighted in this Research Topic—including inflammation, vascular burden, metabolic load, and circadian disruption—are known to influence epigenomic signatures. Incorporating such regulatory layers into multimodal biomarker frameworks may enhance mechanistic interpretation and facilitate personalized risk stratification. Importantly, a global perspective in this context extends beyond the inclusion of multiple biomarker modalities and encompasses variability across populations and health-care systems. Growing evidence suggests that biomarker performance, disease trajectories, and underlying biological mechanisms may differ according to genetic background, environmental exposures, vascular and metabolic burden, and social determinants of health. As blood-based, CSF, imaging, and metabolomic biomarkers move toward broader clinical implementation, their generalizability across diverse and underrepresented populations remains a critical challenge. Recent computational and systems-level studies indicate that models derived primarily from relatively homogeneous cohorts may have limited transferability across settings. Addressing these issues through validation, adaptation, and inclusive implementation strategies will be essential for equitable and scalable dementia biomarker deployment.

In summary, this Research Topic advances a multidimensional view of dementia biomarkers. By integrating findings from blood, CSF, vascular indicators, neuroimaging, proteomics, and metabolomics, the Research Topic offers a foundation for developing diagnostic strategies that reflect the complex, multisystem nature of neurodegeneration.

